# A missing piece in the puzzle: the presence of *Euglossa viridissima* in the Baja California Peninsula (Hymenoptera, Apidae)

**DOI:** 10.3897/zookeys.726.19876

**Published:** 2018-01-02

**Authors:** Armando Falcón-Brindis, Ricardo Ayala, María Luisa Jiménez, Ismael A. Hinojosa-Díaz

**Affiliations:** 1 Laboratorio de Aracnología y Entomología, Centro de Investigaciones Biológicas del Noroeste, S.C. Apartado postal 128, 23090, La Paz, Baja California Sur, México; 2 Estación de Biología Chamela (Sede Colima), Instituto de Biología, Universidad Nacional Autónoma de México (UNAM), Apartado Postal 21, San Patricio, Jalisco, México, C. P. 48980; 3 Departamento de Zoología, Instituto de Biología, Universidad Nacional Autónoma de México (UNAM), Tercer Circuito s/n, Ciudad Universitaria, Copilco, Coyoacán, A.P. 70-153, Ciudad de México, Mexico, C. P. 04510

**Keywords:** Biogeography, Cape Region, oases, orchid bees, neotropics

## Abstract

Orchid bees are a conspicuous component of the neotropical bee fauna, with a few species reaching the northernmost natural distribution for the group in northwestern continental Mexico. Among them, *Euglossa viridissima* Friese is here reported for the first time in the Cape Region of the Baja California peninsula, Mexico, where no species of the group have been found previously. These records are presented, their biogeographical implications discussed, and some interpretations of the local factors that influence the bees is presented.

## Introduction

Under the recent documentation of the decline of local pollinator populations (Biesmeijer et al. 2006, Burkle et al. 2013), it is important to monitor the bee fauna composition at local levels (Potts et al. 2010, Goulson et al. 2015). The discovery of species not previously found in particular areas is part of such endeavors. Isolated new records of species are noteworthy particularly in cases involving taxa of exotic origin, from distant or unrelated biogeographic areas, or when they represent a considerable expansion of their known native range. Orchid bees are well known for their external morphological features such as metallic body coloration and long mouthparts, and also for the peculiar perfume collecting behavior of the males (Dressler 1982, Roubik and Hanson 2004). The Euglossini are the only bees within the corbiculate clade (Apini, Bombini, Euglossini, and Meliponini) that are restricted to the neotropics (Cardinal and Packer 2007, Engel et al. 2009), reaching their northernmost distribution in northwestern Mexico, where at least three species of the around 35 found in the country have been recorded (Búrquez 1997, Gonzalez et al. 2017). Orchid bees are powerful long distance flyers, such that females have been found to fly several kilometers while foraging (Janzen 1971, López-Uribe et al. 2008), and males have been recaptured nearly 100 km away (Pokorny et al. 2015). Some euglossine species have been recently discovered in areas that expand considerably their known native range (Anjos-Silva et al. 2006, Anjos-Silva 2007, 2008, Silva and Rebêlo 2009). A notable example of an introduction to a distant area is *Euglossa dilemma* Bembé & Eltz which was discovered in 2003 in southern Florida, and is now naturalized (Skov and Wiley 2005, Pemberton and Wheeler 2006). This species is a cryptic sibling species of *E. viridissima* Friese from which it was recently split (Eltz et al. 2011). *Euglossa viridissima* occurs from Guatemala throughout southern and central Mexico, being one of the few euglossine species that reach the northwestern continental areas of Mexico (Búrquez 1997, Hinojosa-Díaz et al. 2009) with no previous records (before this work) in the Baja California Peninsula. During the development of a wider faunistic bee survey in the state of Baja California Sur, euglossine bees were first observed in the Cape Region. Here the confirmation of these observations is presented, with first records of *E. viridissima* from the Cape Region of Baja California which represent the first for any euglossine species in the area. The biogeographical implications of these records and local factors that could influence its distribution is briefly discussed.

## Materials and methods

The Cape Region of Baja California Sur (BCS), Mexico, is a biogeographic province with distinctive floristic and faunistic elements (Morrone 2005, Halffter et al. 2008). From a paleogeographic approach, it is considered a big island (200 km from continental Mexico) as it has undergone isolation processes (last vicariance event around 3 MYA) since the peninsula’s origins 5-10 MYA (Brusca and Moore 2013, González-Trujillo et al. 2016), favoring high rates of endemism in the region (Wiggins 1980, Roberts 1989). The vegetation of the region includes low deciduous tropical dry forest communities, xeric scrublands, and ecotones between both. The ecotones mark the delimitation of the Cape Region within the subdivision of the Sonoran desert (Axelrod 1978, Rzedowski 2006). An important component of the vegetation of the area are the oases, which are patches of vegetation associated with fresh water springs, which provide water, food and shelter in the middle of arid conditions of the peninsula (Arriaga and Rodríguez-Estrella 1997).

Sampling was carried out from May to November 2016 at 14 localities in the Cape Region (Table 1). Using insect nets the sampling of blooming areas was emphasized, specifically those of *Tecoma stans*. Additionally, chemical attractants (eugenol and eucalyptus oil) were tested intending to collect male bees in those localities where activity of the orchid bees was thought to be more likely, that is, San Bartolo, Santiago, and Todos Santos. The baited traps consisted of 600 ml plastic bottles following protocols used in South America (Sydney and Gonçalves 2015) arranged in 100 m lineal transects (ten traps per transect, 10m separation among individual traps). On each locality mentioned above one transect was set, with the traps staying for 24 h in every case.

**Table 1.** Sampled localities in the Cape Region, Baja California Sur State, Mexico.

Locality	Coordinates	Elevation (m)
Cabo Pulmo	23°26'06.70"N, 109°25'58.00"W	24
El Triunfo	23°48'12.90"N, 110°06'31.60"W	482
Las Cuevas	23°30'52.50"N, 109°41'23.50"W	125
La Ribera	23°33'21.80"N, 109°33'00.90"W	35
Los Planes	23°58'41.70"N, 109°58'18.20"W	24
Melitón Albáñez	23°38'26.80"N, 110°17'02.00"W	163
Santiago	23°28'37.90"N, 109°42'36.70"W	113
San Antonio de la Sierra	23°40'22.40"N, 109°55'51.10"W	947
San Bartolo	23°44'18.07"N, 109°50'48.85"W	389
San Dionisio	23°32'16.80"N, 109°47'53.60"W	371
Santuario de los cactus	23°44'45.10"N, 110°06'43.60"W	435
Sierra de la Laguna	23°33'06.60"N, 109°59'07.00"W	1752
San Pedrito	23°23'23.40"N, 110°12'26.90"W	17
Todos Santos	23°26'57.53"N, 110°13'35.35"W	32

## Results

**Figures 1–4.**
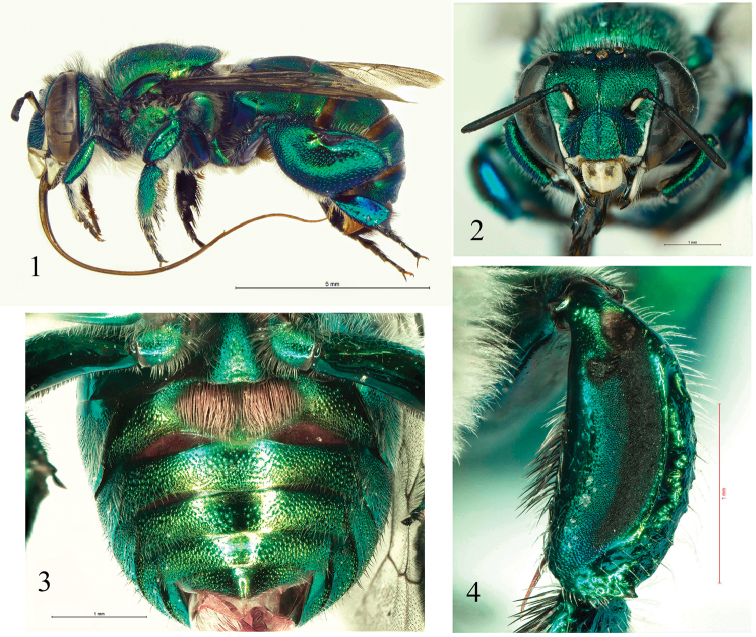
Male details of *E. viridissima* found in Baja California Sur State. **1** Habitus (lateral view) **2** Facial aspect **3** Ventral view of metasoma **4** Mesotibia.

**Figure 5.**
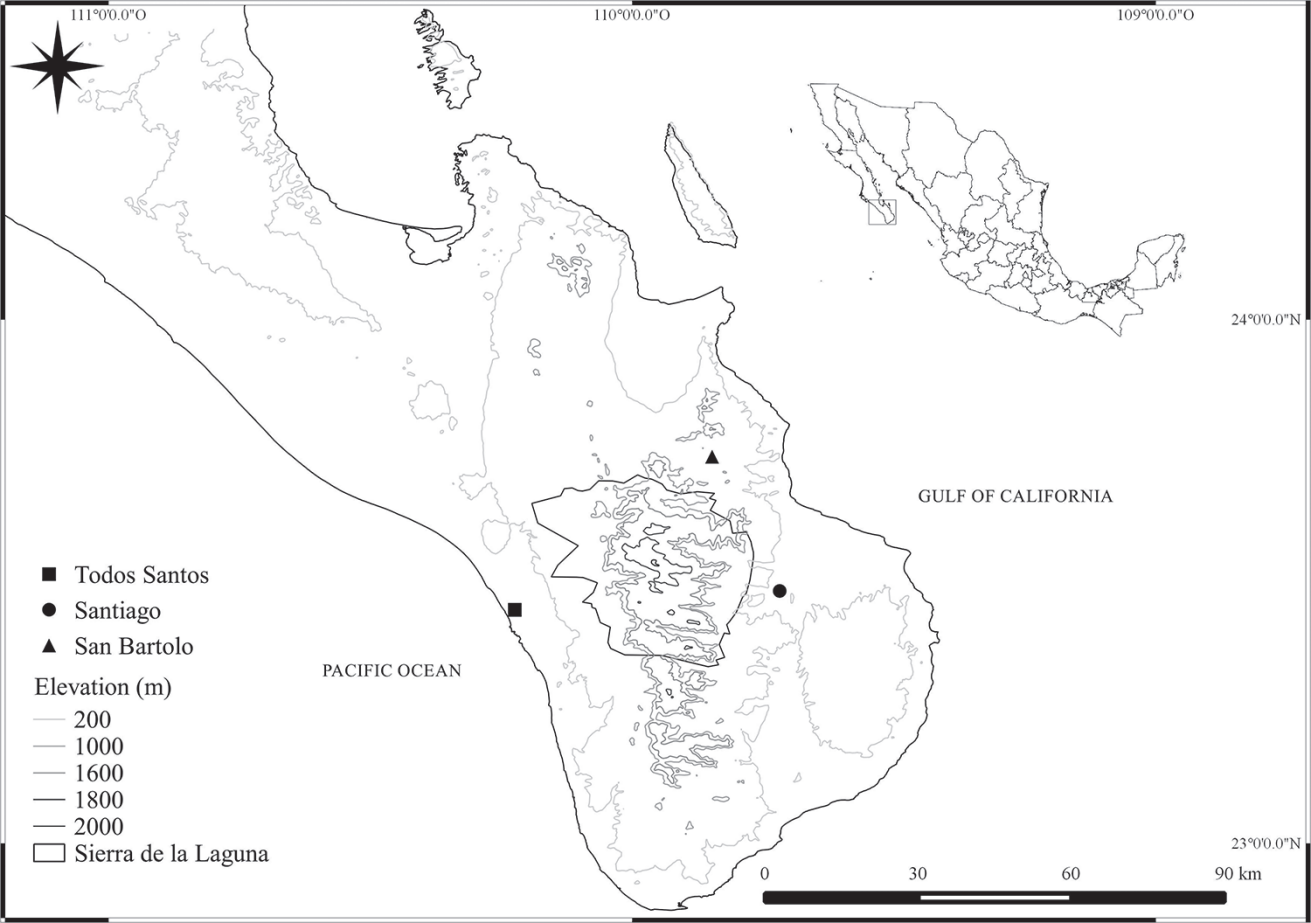
Localities in which was registered the presence of *E. viridissima* in the Cape Region. Biogeographic Provinces map from CONABIO (1997).

Of the 14 Cape Region localities sampled, *E. viridissima* (Figs 1–4) was found only in Todos Santos on the Pacific slope, Santiago and San Bartolo on the Gulf of California slope (Fig. 5).

In total, 33 specimens (19 males, 14 females) of *E. viridissima* were collected. Per locality, 30 specimens (17 males, 13 females) came from Todos Santos; all were collected in August, a single female from San Bartolo collected in April, and two males from Santiago collected in October. Most of the specimens were caught in oases vegetation (96.7%). All the bees were captured while visiting flowers of *Tecoma stans*. The male specimens of *E. viridissima* were not attracted to the traps baited with chemical attractants.

Voucher specimens are deposited into in the entomological collection at the CIBNOR (La Paz, Mexico).

## Discussion

The finding of *Euglossa viridissima* as the first record of an orchid bee species on the Baja California peninsula has several implications. Biogeographically, *E. viridissima* has the northernmost natural distribution within Euglosines (Búrquez 1999, Roubik and Hanson 2004, Hinojosa-Díaz et al. 2009, Ramírez et al. 2010). In addition, this finding represents both a new and distinctive biogeographic area to the distribution of the species, and a new Neotropical bee record to the mainly Nearctic peninsula (Morrone 2005). Before our records of *E. viridissima* in the Cape Region of the peninsula, no other euglossines had been reported from there (Ayala et al. 1996, Moure et al. 2007, Ascher and Pickering 2017).

The Cape Region of the Baja California Peninsula is separated from the nearest Mexican mainland by the Gulf of California by around 200 km, much further than the nearly 100 km that a male *E. viridissima* was registered to fly when attracted to a bait in the Yucatán Peninsula (Pokorny et al. 2015). While most of the South American expansion records are likely due to the bees own dispersal capabilities (Anjos-Silva et al. 2006, Anjos-Silva 2007, 2008, Silva and Rebêlo 2009, Silva et al. 2013, Martins et al. 2016), the *E. viridissima* records from Baja California are unlikely to have gotten there by long distance migration. Alternatively, these bees are cavity nesters (May-Itzá et al. 2014), making it possible that occupied nests would survive the carrying from the continental lands to the peninsula. Also possible is that they were brought over accidentally along with normal commerce. The morphological conspicuousness of these bees (Figs 1–4) makes it hard to think that they have been in the area for long with no one noticing them before, so our best guess is that as these bees are a relatively recent arrival.

*Euglossa viridissima* appears to be well-established on Baja California, since the three sampled points (Fig. 5) are rather spread over the Cape Region and both sexes were relatively abundant at Todos Santos. However, the potential distribution modeled by Hinojosa-Díaz et al. (2009) predicted there was not suitable habitat anywhere in the peninsula of Baja California for *E. viridissima*, as understood then, but for *Eulaema polychroma*, one of the other orchid bee species reaching the northwestern continental areas of Mexico.

Our floristic observations of the host plant differ from Arriaga and León de la Luz (1989), who found *T. stans* as a predominant species in some patches at the Pacific hills compared to the Gulf slope. Weather conditions are complex when comparing these two slopes in the Cape Region. Roberts (1989) mentioned that climatic and physiographic conditions make the Gulf slope more humid and hotter than the west side of the peninsula. However, Díaz and Troyo (1997) found drier and hotter oases in the east slope influenced by local phenomena. Arriaga and León de la Luz (1989) explained higher plant richness at the Pacific slope because its cooler and more humid conditions compared to the Gulf slope.

It is possible that abiotic factors (e.g. moisture, temperature) have more of an effect on populations than food availability. We do not discard the possibility that natural enemies also play an important role on these boundaries (e.g. more humid places increase likelihood to fungus attacks on immature stages). The new finding of *E. viridissima* at the Cape Region highlights its biological relevance as an important element of the Neotropical area. In addition, since the oases of the Baja California peninsula are shaped by different factors such as water availability, type of soil, geographical position, and degree of anthropogenic disturbance, the biological communities may respond to such insular-like conditions, presenting variation in structure and abundance (Jiménez et al. 2015, Arriaga and Rodríguez-Estrella 1997). Furthermore, considering the about 21 species of orchids restricted to some deep valleys or higher elevations (>600 m) in Sierra de La Laguna, BCS (Medel-Narváez pers. com. Jan. 20^th^ 2017), it makes conceivable to think of possible euglossines-plant interactions, but also to find specific relationships with endemic orchid species. However, further research on these subjects is encouraged.

Overall, the records of *E. viridissima* in the Cape Region of the Baja California peninsula represent an important piece of information regarding these bee’s distribution and likely dispersal or ecological capabilities. They also bring the opportunity to stress the need to sample the local bee faunas, in a time when pollinators are known to be declining in different parts of the world.
